# Association of hyperuricemia with apolipoprotein AI and atherogenic index of plasma in healthy Chinese people: a cross-sectional study

**DOI:** 10.1186/s12872-022-02810-7

**Published:** 2022-08-15

**Authors:** Yan Duan, Xiaona Chang, Xiaoyu Ding, Yu An, Guang Wang, Jia Liu

**Affiliations:** Department of Endocrinology, Beijing Chao-yang Hospital, Capital Medical University, Beijing, 100020 China

**Keywords:** Hyperuricemia, AIP, Apo AI

## Abstract

**Background:**

The atherogenic index of plasma (AIP) is a predictor for cardiovascular diseases (CVD), while hyperuricemia is an independent risk factor for a variety of CVD. Apolipoprotein AI has been found to be a protective factor for CVD. However, the role of APO AI in the association between plasma uric acid and AIP among healthy Chinese people needs to be further explored.

**Aims:**

To evaluate the relationship between blood uric acid and AIP level in healthy Chinese people. To evaluate the relationship between blood uric acid and Apolipoprotein AI in healthy Chinese people.

**Method:**

A total of 3501 normal and healthy subjects who had physical examinations were divided into the hyperuricemia (HUA) group and the normouricemia (NUA) group.

**Result:**

The AIP of HUA group was significantly higher than that of NUA group [0.17±0.30 vs. −0.08±0.29]. Apo AI (1.33 ± 0.21 vs. 1.47 ± 0.26 g/l) and HDL-c (1.12 ± 0.27 vs. 1.36 ± 0.33 mmol/l) were significantly lower in the HUA group than in the NUA group. LDL-C (2.81 ± 0.77 vs. 2.69 ± 0.73 mmol/l), Apo B (0.96 ± 0.20 vs. 0.89 ± 0.20 g/l), FBG (5.48 ± 0.48 vs. 5.36 ± 0.48 mmol/l) and HOMA-IR [2.75 (1.92–3.91) vs. 2.18 (1.50–3.12)] was significantly higher in HAU group than the NUA group. Increases in plasma UA were associated with increases in AIP (β = 0.307, *p* < 0.01) and decreases in Apo AI (β =  − 0.236, *p* < 0.01).

**Conclusion:**

Hyperuricemia is an independent risk factor for high AIP level. Inhibition of Apolipoprotein AI may be one of the mechanisms of UA which is involved in the progression of cardiovascular disease.

## Introduction

A large number of studies have shown that hyperuricemia is closely related to cardiovascular disease. It is known that hyperuricemia is associated with a significant increased risk of hypertension, coronary heart disease (CHD), and congestive heart failure (CHF) [1–3]. Additionally, another study found that serum uric acid is an independent predictor for cardiovascular disease-related death, including chronic, acute, and subacute forms of CHF, CHD and stroke [4, 5]. These are all related to the deposition of urate crystals in the vascular endothelium and the dissolution of urate promotes lipid peroxidation, which increase oxidative stress and inflammatory response and lead to vascular endothelial dysfunction [6, 7].

Dyslipidemia is also an independent risk factor for cardiovascular disease. High-density lipoprotein cholesterol (HDL-C) is a cardiovascular protective factor [8]. Apolipoprotein(apo)A-I is the principal protein of HDL-C [9]. Previous studies found that hyperuricemia is closely related to HDL-C and Apo AI. Hyperuricemia is often accompanied by abnormal lipid metabolism, including low HDL-C level [10]. Moreover, it was found that the ratio of apolipoprotein-B to AI are strongly associated with serum uric acid levels in US people [10].

AIP was calculated as lg (TG (mmol)/L/HDL (mmol/L)), which was found to be a powerful indicator of CVD among different population [11–14]. Another study in Indonesia showed that low AIP predicted a decrease in all-cause mortality of hospitalized patients with acute myocardial infarction [15]. It has been proved that AIP is associated with smaller LDL particles [16] and increased esterification rate of HDL [17]. Recently, several studies have shown that AIP is related to other metabolic indexes besides Lipid metabolism. Akbas et al. [18] reported that plasma UA was independently positively correlated with AIP in patients with diabetes mellitus. However, the relationship between apolipoprotein, uric acid and AIP has not been confirmed in a large number of Chinese people.

Furthermore, although there are several studies which focus on the relationship between uric acid and lipid metabolism, the participants with hyperuricemia in most of the previous studies had comorbidities such as diabetes, hypertension, or cardiovascular diseases. There may be mutual interference between the diseases, which therefore makes clarifying the simple relationships a difficult task. In the present study, we examined the relationship between hyperuricemia and Apo AI and AIP in normal and healthy Chinese subjects.

## Methods

### Design and participants

A total of 3501 healthy individuals aged over 20 years and under 80 years were enrolled in this study. All participants had undergone a routine physical examination at Beijing Chao-yang Hospital Affiliated to Capital Medical University from March 2012 to October 2014. Individuals with hypertension, diabetes, pre-diabetes, cancer, liver or renal function impairment, coronary artery disease, or systemic inflammatory disease were excluded. Participants who took lipid-lowering or uric-acid-lowering agents were also excluded. HUA was defined by the plasma UA level ≥ 420 mol/L in men and ≥ 360 mol/L in women [19]. We divided the participants into two groups: the NUA group (subjects without hyperuricemia) and the HUA group (subjects with hyperuricemia). The protocol of the study was approved by the Ethics Committee of Beijing Chao-yang Hospital Affiliated to Capital Medical University.

### Physical and biochemical measurement

Height, weight, systolic blood pressure (SBP), and diastolic blood pressure (DBP) were measured. BMI was calculated as weight (kg)/height (m)^2^.

Blood samples were collected from the vein after a 12-h fasting period. Each sample from participants was stored at − 80 °C. HDL-C and low-density lipoprotein cholesterol (LDL-c) were measured using the direct assay on an autoanalyzer (Hitachi 7170). The levels of Triglyceride (TG), total cholesterol (TC) and Serum uric acid (UA) were measured by glycerol lipase oxidase reaction, enzymatic cholesterol oxidase method on a Hitachi 7170 autoanalyzer and enzymatic assay, respectively. Immune turbidimetry was utilized to analyze the immune turbidimetry ApoAI and apolipoprotein B (Apo B). The concentration of fasting insulin (FINS) and fasting blood glucose (FBG) was measured at the central chemistry laboratory in Beijing Chao-yang Hospital.

AIP was calculated as lg (TG (mmol)/L/HDL (mmol/L)).

Homeostasis model assessment of β-cell function (HOMA-β) was calculated as 20*FIN/ (FBG-3.5).

Homeostasis model assessment of insulin resistance (HOMA-IR) was calculated as FINS (μIU/mL) * FBG (mmol/L)/22.5.

### Statistical methods

All statistical analyses in this study were performed using SPSS version 21.0. Continuous variables with normal distributions were shown as a mean ± standard deviation (SD), while continuous variables with skewed distributions were expressed as a median with upper and lower quartiles. The Student *t*-test and non-parametric test were applied to analyze the differences between the groups. The continual variables of abnormal distribution were expressed as the median of upper and lower quartiles and analyzed by a nonparametric test. Discontinuous variables were given as percentages and analyzed by chi-square test. Pearson’s correlation analysis was performed to measure the linear correlation between the variables that was normally distributed. Linear regression and logistic regression analysis were elaborated to further explore the association between blood uric acid and Apolipoprotein AI level or other metabolism indexes. Statistical significance was defined as *p* < 0.05.

## Results

### Clinical characteristics of the HUA and NUA group

**Fig. 1:**
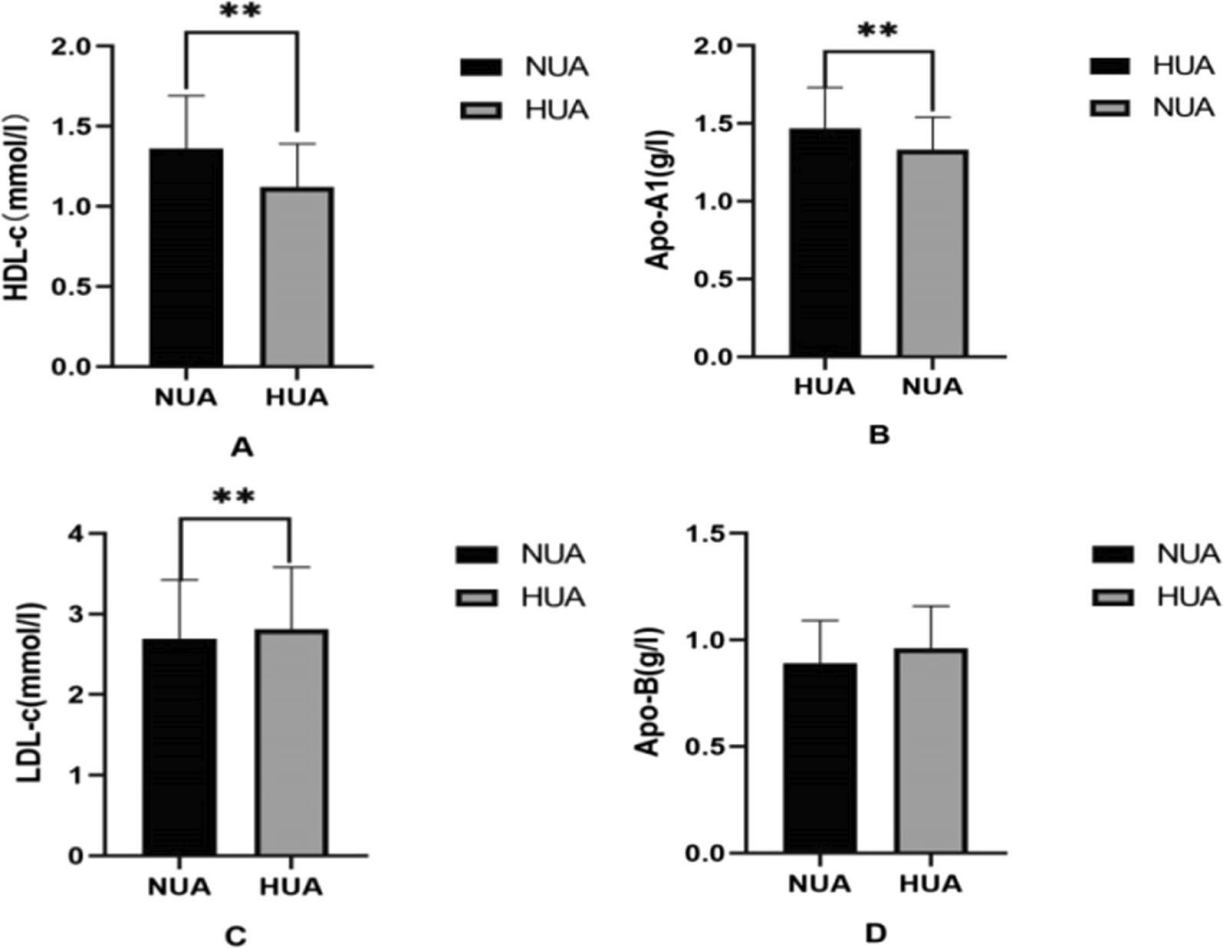
Comparison of lipid metabolism indices between HUA and NUA group. Bar graph of Lipid metabolism indices (**A** HDL-c; **B** Apo AI; **C** LDL-c; **D** Apo-B) mean value with SD intervals between NUA and HUA groups. *p < 0.05, **p < 0.01. *UA* uric acid, *HDL-c* high-density lipoprotein cholesterol, *Apo AI* apolipoprotein A1, *LDL-c* low-density lipoprotein cholesterol, *Apo B* apolipoprotein B

**Fig. 2:**
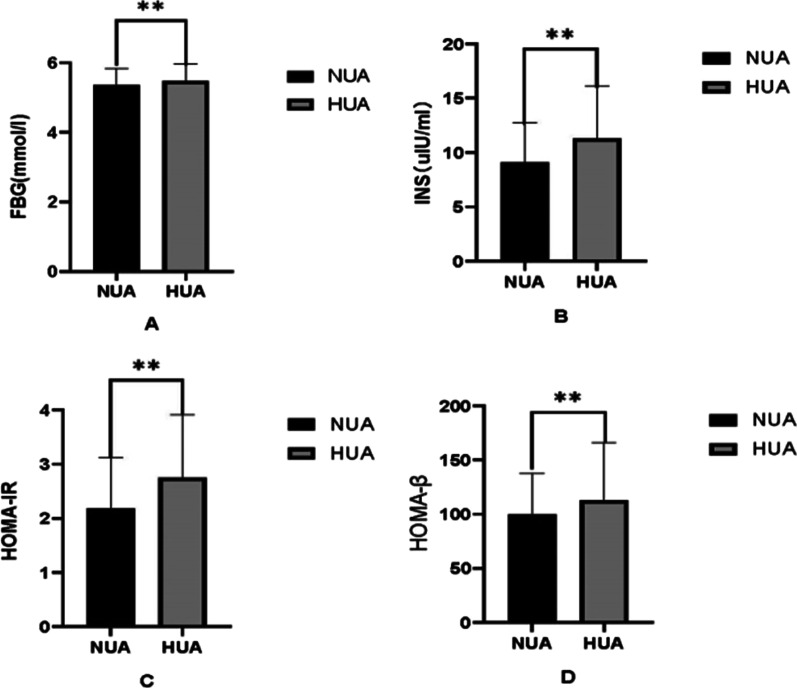
Comparison of glucose metabolism indices between HUA and NUA group. Bar graph of glucose metabolism indices (**A** FBG; **B** INS; **C** HOMA-IR; **D** HOMA-β) mean value with SD intervals or median with quartiles between NUA and HUA groups. *p < 0.05, **p < 0.01. *FBG* Fasting blood glucose, *INS* insulin, *HOMA-IR* homeostasis model assessment of insulin resistance, *HOMA-β* homeostasis model assessment of β-cell function

The clinical characteristics of HUA group and NUA group are shown in Table 1, from which it can be seen that there were 2868 subjects in NUA group and 633 subjects in HUA group. The proportion of males in the HUA group was significantly higher than that in the NUA group. BMI, SBP and DBP increased significantly in the HUA groups, more than the NUA groups (*p*<0.01). The level of AIP was found to be significantly higher in the HUA group than in the NUA group. For lipid metabolism comparison, TG, LDL-c and Apo B were significantly increased in HUA participants, and HDL-C and Apo AI were lower than those in the NUA group (*p* < 0.01) (Fig. 1). Furthermore, FBG and FIN concentrations were significantly higher in the HUA group compared with the HUA group due to higher HOMA-β and HOMA-IR (*p* < 0.01) (Fig. 2). AIP was found at higher values in the HUA group than the NUA group.

**Table 1:** Comparison of Characteristics between NUA and HUA

Parameters	NUA group (n = 2868)	HUA group (n = 633)	*p* Value
Age, y	45.2 ± 11.6	41.5 ± 11.9	< 0.01
Gender, male%	41.00	95.10	< 0.01
BMI, kg/m^2^	23.49 ± 2.85	25.42 ± 2.47	< 0.01
SBP, mmHg	118.4 ± 11.9	122.7 ± 10.6	< 0.01
DBP, mmHg	71.5 ± 8.8	75.0 ± 8.5	< 0.01
TG, mmol/l	1.05 (0.76–1.51)	1.57 (1.12–2.32)	< 0.01
LDL-C, mmol/l	2.69 ± 0.73	2.81 ± 0.77	< 0.01
HDL-C, mmol/l	1.36 ± 0.33	1.12 ± 0.27	< 0.01
FBG, mmol/l	5.36 ± 0.48	5.48 ± 0.48	< 0.01
FINS, uIU/ml	9.11 (6.41–12.74)	11.31 (8.01–16.12)	< 0.01
UA, umol/l	300.88 ± 64.71	474.13 ± 55.47	< 0.01
CRE, umol/l	61.73 ± 12.62	76.65 ± 10.82	< 0.01
AIP	− 0.08 ± 0.29	0.17 ± 0.30	< 0.01

*BMI* body mass index, *SBP* systolic blood pressure, *DBP* diastolic blood pressure, *UA* uric acid, *TG* triglyceride, *HDL-c* high-density lipoprotein cholesterol, *LDL-c* low-density lipoprotein cholesterol, *FBG* fasting blood glucose, *FINS* fasting insulin, *CRE* creatinine, *AIP* atherogenic index of plasma

### Correlation between UA and AIP

**Fig. 3:**
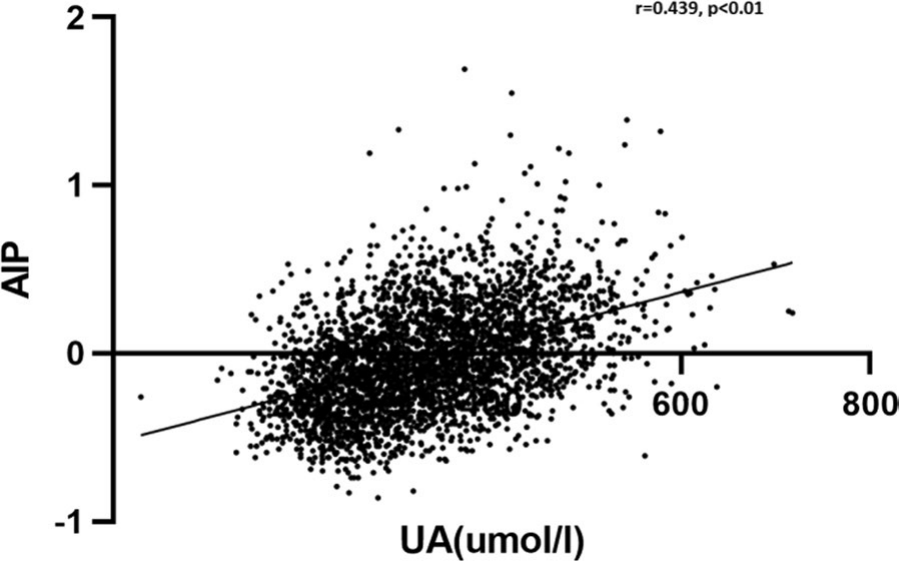
The correlation between plasma UA and AIP. Correlation analysis was used to prove the linear relationship between plasma UA and AIP. Plasma UA was positively correlated with AIP

UA was positively correlated with AIP, after applying linear correlation analysis (r = 0.439, *p* < 0.01) (Fig. 3). To further examine the correlation between UA and AIP, multiple linear regression analysis was performed. After adjustment for age, BMI, SBP, and FBG, there was still a significant relationship between the increase in UA and the increase in AIP value (β = 0.307, *p* < 0.01).

### Correlation between UA and Apo AI or other metabolic indexes

**Fig. 4:**
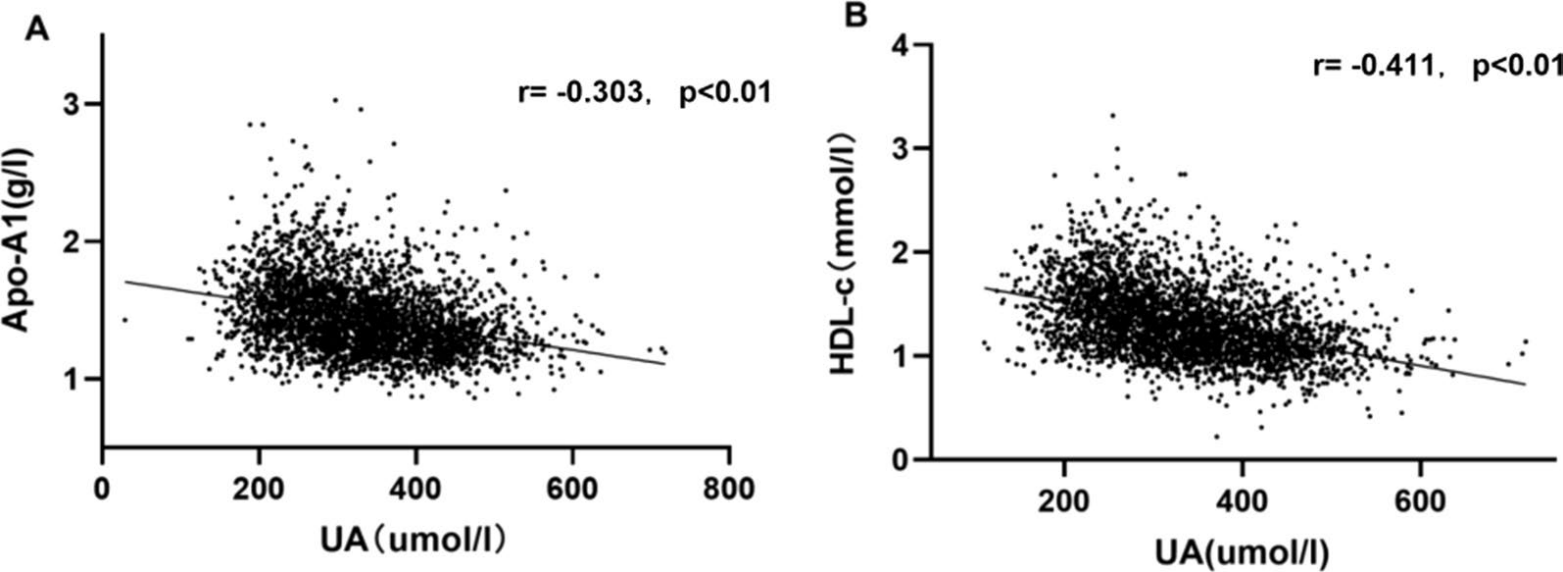
Correlation between plasma UA and the levels of Apo AI and HDL-c. Correlation analysis was used to prove the linear relationship between plasma UA and the levels of Apo AI (**A**) and HDL-c (**B**). Plasma UA was negatively correlated with Apo AI and HDL-C

UA was inversely correlated with Apo AI and HDL-c, after applying linear correlation analysis (Apo AI: r = −0.303, *p* < 0.01; HDL-c: r = −0.411, *p* < 0.01) (Figure 4). Multiple linear regression analysis was used to further prove the correlation between UA and other metabolic indexes. After adjusting for age, BMI and SBP, FBG was positively associated with UA (r = 0.046, *p* < 0.05) and Apo AI was inversely associated with UA significantly (r = −0.236, *p* < 0.01). Also, it was found that HDL-C was inversely associated with UA (β = −0.289, *p* < 0.01)

To study whether HDL participates in the relationship between Apo AI and UA, another multivariate logic analysis model was set up. UA was used as dependent variable. Age, Gender, BMI, SBP, AIP, FBG, CRE, Apo AI and Apo B were used as independent variables. In this model, AIP was positively associated with UA [odds ratio (OR): 5.449; 95% confidence interval (CI): 3.408–8.714)]. Apo AI has no significant correlation with UA (*p* > 0.05) (Table2).

**Table 2:** Multivariate logistic regression analyzed factors associated with UA

	OR	95%CI	*p* Value
Age	0.967	0.956–0.978	< 0.001
SBP	1.006	0.995–1.017	0.298
BMI	1.095	1.042–1.150	< 0.001
Male	6.232	3.841–10.112	< 0.001
Apo B	3.074	1.605–5.888	0.001
Apo AI	0.980	0.538–1.785	0.946
FBG	0.984	0.769–1.259	0.899
CRE	1.056	1.043–1.069	< 0.001
AIP	5.449	3.408–8.714	< 0.001

Compared to non hyperuricemia. Variables included in the model were Age, male, BMI, SBP, AIP, FBG, CRE, Apo AI and Apo B. The Pseudo R2 (McFadden R2) was 0.316. The *p* value of likelihood ratio test was < 0.01

*BMI* body mass index, *FBG* fasting blood glucose, *SBP* systolic blood pressure, *CRE* creatinine, *AIP* atherogenic index of plasma, *Apo AI* apolipoprotein A1, cholesterol, *Apo B* apolipoprotein B, *FBG* fasting blood glucose

## Discussion

Presently, many studies have found that HUA is closely related to cardiovascular disease. For example, it can affect vascular endothelium, increase the risk of hypertension [20, 21], heart failure [22], increase the risk of cardiovascular disease, including coronary heart disease [23], and myocardial infarction [5, 24]. Although many studies on the mechanism partly explain the relationship between UA and cardiovascular disease, it is not completely clear. Moreover, hyperuricemia is often accompanied by other diseases. At present, among a large number of people engaged in the research on uric acid and metabolic or cardiovascular diseases, there are often other metabolic or cardiovascular comorbidities or even metabolic syndrome. It is difficult to purely study the relationship between UA and specific risk factors, or protective factors of cardiovascular disease. This study is characterized by the fact that it is conducted in a healthy population without diabetes, hypertension, or metabolic syndrome, and to clarify the relationship between UA and AIP and Apo AI. The result plays a certain role in the occurrence and development mechanism of UA in cardiovascular disease.

The present study explored the association between UA and Apo AI in healthy people without diabetes, hypertension or metabolic syndrome. Apo AI is one of the cardiovascular protective factors [25]. We found that the Apo AI and HDL-C in the HUA group were lower than that in the NUA group. In further analysis, HUA was found to be independently associated with decreased plasma Apolipoprotein AI and HDL-C. The results may suggest that hyperuricemia may promote the progress of the cardiovascular disease by reducing the levels of Apo AI and HDL-C. Other previous studies are similar to our study [26]. Kuwabara et al. [21] conducted a prospective study on Japanese patients with hyperuricemia but without typical symptoms and other complications. The study found that after a five-year follow-up the incidence of cardiovascular related metabolic disorders, such as abnormal lipid metabolism and hypertension, in the asymptomatic HUA group was significantly higher than that in the normal UA group. A sub-analysis of the NHANES III study also found that triglycerides to HDL-C and Apo-B to Apo AI were linearly positively associated with uric acid levels. However, in contrast with our study, the participants included patients with diabetes, hypertension and other complications, and no significant correlation between Apo AI and UA after adjusting for related factors [10].

Presently, the mechanism of the interaction between uric acid with HDL-C and Apo AI is not completely clear. Animal studies have found that high uric acid can reduce the level of phospholipids of HDL subclasses. Also induce the increase of fractional catabolic rate (FCR) significantly, resulting in decreased HDL-C and Apo AI levels [27, 28]. Other studies have found that high UA is also closely related to small and dense HDL-C. At the same time, HDL-C volume is negatively correlated with fibrinogen concentration [29], and HUA was negatively correlated with a large HDL-C level [23], which may present a mechanism that contributes to arteriosclerosis. There are different relationships between HUA and different subclasses of HDL-C [30], HUA and HDL2, which are associated with alcohol consumption [31], waist circumference, smoking, and exercise had a negative correlation. However, HUA and HDL3 which only associated with alcohol consumption had a positive correlation. Furthermore, most studies only studied the relationship between HDL and UA; the mechanism of the interaction between Apo AI and UA needs to be further explored.

Additionally, results from this study suggested that other metabolic parameters, including FBG and HOMA-IR, were associated with UA rather than lipoprotein. Comparable with this study, many studies have found that HUA is closely related to insulin resistance and hyperglycemia [32–34]. Long term follow-up studies confirmed that hyperuricemia is an indicator for predicting abnormal glucose metabolism. Krishnan et al. [35] conducted a 15-year follow-up study on young people without diabetes. They found that the risk of developing diabetes, insulin resistance (IR) and prediabetes in the HUA group was significantly higher than that in the non-hyperuricemia group. A 5.3-year follow-up study of the Chinese population also confirmed that HUA and is closely related to the development of hypertension [36]. The mechanisms are complex; uric acid can reduce IR by promoting mitochondrial oxidative stress and NO bioavailability [37, 38]. However, hypouricemic drugs such as Allopurinol can reduce uric acid, improve insulin resistance, and systemic inflammation in patients with hyperuricemia [39]. Adversely, IR can induce hyperuricemia by inhibiting uric acid excretion through increasing renal tubular sodium reabsorption [40].

Compared to other lipid metabolism indexes, AIP was proven as the strongest predictor for coronary artery disease [11]. A recent study has found that AIP is independently related to the rapid progress of plaque in coronary artery of patients with CAD, thus affecting the progress rate of CAD [41]. Our result showed that UA was positively correlated with AIP in multiple linear regression analysis, which was similar to the research in the population of Northeast China conducted by Chang et al. [42]. However, the research included diabetes, cardiovascular disease and other complications of the crowd, and did not measure or analyze lipoproteins level in detail. Another study in India found AIP was positively associated with serum UA, TG, and HDL-C [43]. Which differed from the present study, females were not incorporated and APO AI was not measured. In the current study, the relationships among uric acid and AIP and Apo AI were analyzed. We hypothesize that decreased levels of Apo AI and HDL is one of the mechanisms involved in the deterioration of AIP caused by hyperuricemia.

The limitations of our study include that fact that this is a cross-sectional study, and it is impossible to determine the causal relationship. A prospective study can be carried out next to further explore the relationship between UA, Apo AI and cardiovascular disease. This study is calculated upon the data of a small number of subjects who received health examinations, which had selection bias, and larger scale studies will be carried out in the future.

## Conclusion

In conclusion, hyperuricemia was an independent risk for increased AIP levels and associated with decreased plasma Apolipoprotein AI independently. Inhibition of Apolipoprotein AI may be one of the mechanisms of UA involved in the progression of cardiovascular disease.

## Data Availability

The datasets used and analyzed during the current study are available from the corresponding author on reasonable request.
